# Requirement for Abasic Endonuclease Gene Homologues in *Arabidopsis* Seed Development

**DOI:** 10.1371/journal.pone.0004297

**Published:** 2009-01-27

**Authors:** Terence M. Murphy, Mark Belmonte, Stephanie Shu, Anne B. Britt, James Hatteroth

**Affiliations:** Section of Plant Biology, University of California Davis, Davis, California, United States of America; Purdue University, United States of America

## Abstract

*Arabidopsis thaliana* has three genes, *Ape1L*, *Ape2*, and *Arp*, that show homology to abasic (apurinic/apyrimidinic) endonuclease genes of bacterial, yeast, or animal cells. In bacteria, yeast, and animals, abasic endonucleases function in base excision repair of oxidized and other modified DNA bases. Here we report that plants with knock-out mutations in any one of *Ape1L*, *Ape2*, or *Arp* show no apparent differences from wild type in growth rate, growth habit, and fertility. However, coincident knock-out mutations in *Ape1L* and *Ape2* are lethal and lead to abortion of developing embryos. Mutations of *Arp* are not deleterious, even in combination with one of the other two mutations. The results are consistent with the interpretation that the process of base excision repair, involving at least one intact copy of *Ape1L* or *Ape2*, is required in the process of embryogenesis.

## Introduction

Base excision repair is essential for genetic integrity in bacteria, fungi, and animals. This process removes and replaces DNA bases when they have been oxidized or alkylated. In plants, many of the genes of the base excision repair pathway have been identified [1], [2]. However, the function of the pathway in plants has been more difficult to demonstrate. For instance, the first step in base excision repair is the removal of a base by a glycosylase. The most prevalent type of damage that is repaired by the base excision repair pathway is 7,8-dihydro-8-oxoguanine (8-oxo-G) in DNA [3], and plants possess the homologs of genes for two enzymes, oxoguanine glycosylase (OGG) and formamidopyrimidine-DNA glycosylase (FPG), that remove the 8-oxo-G. There is so far no evidence to show that these enzymes are essential: single and double knock-out mutations of the two genes that code these glycosylase enzymes in *Arabidopsis thaliana* do not increase the sensitivity of the plants to oxidative stresses or reduce the viability of seed [4]. There are, however, other types of base changes repaired by the base excision repair pathway, suggesting that the loss of a downstream function in the pathway might have more serious effects.

The second step in the base excision repair pathway is scission of the sugar-phosphate backbone of the DNA at the site where the base was removed. This may be catalyzed coincident with base removal by certain dual-function glycosylases, or it may be catalyzed by a separate abasic (apurinic/apyrimidinic, Ap) endonuclease [5]. *Arabidopsis* has three genes, *Ape1L*, *Ape2*, and *Arp*, that show homology to human abasic endonuclease genes. Only the expression product of *Arp* has been described [6]. The *Arp* gene product has a class II abasic endonuclease activity, but it also has a redox function that stimulates the DNA-binding activity of the human transcription factors Fos and Jun.

To further characterize the function of the base excision repair pathway in plants, we have located and combined insertion mutations of the three abasic endonuclease genes. There was no apparent phenotype associated with the removal of the *Arp* gene, either alone or in combination with *Ape1L* or *Ape2*. However, the results indicate an essential role for the *Ape1L* and *Ape2* gene products in the development of the embryo, the stage of seed development that follows fertilization. This role may reflect the need for repair of DNA damage in the embryo; alternatively, it may represent a need for modification of DNA to activate expression of specific genes.

## Materials and Methods

### Sources of mutant strains

**Figure 1:**
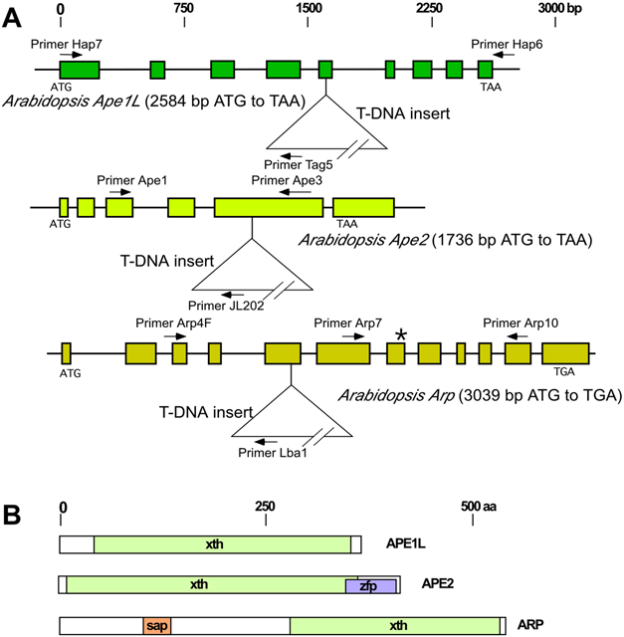
A. Relative sizes and structures of the genes for abasic endonucleases *Ape1L*, *Ape2*, and *Arp*. Diagrams show intron/exon structures, the positions of primers used to assay the identities of alleles, the locations of T-DNA inserts and, for *Arp*, the mis-sense mutation (*). For *Ape2*, only the predicted coding regions of gene model At4g36050.1 are shown. B. Relative sizes and predicted domains of the APE1L, APE2, and ARP proteins. xth, Xth exodeoxyribonuclease III; sap, DNA binding; zfp, GRF-type zinc-finger protein.

The *Arabidopsis Ape1L* gene (At3g48425) was identified by its homology to the human *Ape1* gene. The gene sequence and mRNA sequence have been described in Genbank accessions AL049659 and BX823375, respectively (Fig. 1A). T-DNA insertion mutants of this gene were obtained from the INRA and Salk collections (INRA Flag 240B06; Salk 024194). The Salk insertion was found to be 89 bases upstream from the translation start site, whereas the INRA insertion was in the fifth exon (position 17944461). The INRA strain, here termed *ape1L-1*, was used for all the experiments described in the present study. The presence of the insertion was tested by PCR using a combination of three primers: *Ape1L* forward (“HAP7”): 5′CCCTGCCTTTCGCCGGAAAAGCGT3′; *Ape1L* reverse (“HAP6”): 5′TAACCTCAATCAAATCTTCAATGCATCTC3′; T-DNA left border (“TAG5”): 5′CTACAAATTGCCTTTTCTTATCGAC3′. The wild-type allele gave a band at 2645 bp; the insertion mutant allele gave a band at ca 1700 bp. Alternatively, the wild type allele was identified using 5′CTCCTTCGGGTGAAAAATGA3′ (“HAP2”) as forward primer and 5′CTTACCTCTTTGTTTGGAGG3′ (“HAP10”) as reverse primer; and the insertion allele was tested in separate reactions using TAG5 and HAP10.

The *Arabidopsis Ape2* gene (TAIR At4g36050; Fig. 1A) was identified by its homology to the human *Ape2* gene. RNA from adult plants was extracted by standard methods (see below); cDNA was prepared (see below) and sequenced by RT-PCR, using primers based on the gene sequence. There was clear evidence for gene model At4g36050.1 (Fig. 1) and no consistent evidence for the longer gene model At4g36050.2. The results were submitted to GenBank (accession EU517563). A mutant with a T-DNA insertion in the fourth exon (chromosome 4, position 17052961), here termed *ape2-1*, was obtained from the University of Wisconsin Arabidopsis Knock-out Facility [7]. The presence of the insertion was tested by PCR using the three primers, *Ape2* forward (“Ape1”) 5′TTGATCATATCTTAGTTGCTGG3′; *Ape2* reverse (“Ape3”) 5′GAAACATTCGGTTCTGTGATGC3′; and JL202 5′CATTTTATAATAACGCTGCGGACATCTAC3′.

The *Arp* gene (TAIR At2g41460; Fig. 1A) was identified using primers to conserved regions of the human REF/HAP endonuclease and other endonucleases [6]. RNA from adult plants was extracted by standard methods; cDNA was prepared and sequenced; and the results were submitted to GenBank (accession EU517564; Fig. 1). A mutant, here termed *arp-1*, with an R354W substitution in a conserved site was obtained through the TILLING procedure (Fig. 1)[8], and strains containing the mutation were identified by treating a PCR product, synthesized with forward (“Arp7”, 5′ATTGTTGAAGTTTGAGAGCTTC3′) and reverse (“Arp10”, 5′TAAGTGTAACCGACAACACCAG3′) primers, with *NlaIII* restriction enzyme. The wild-type gene gave a band at 425 bp; the mutant gene gave two bands, at 297 and 128 bp. A second mutant, here named *arp-2*, with a T-DNA insertion in the fifth exon (Salk 021478, insert in chromosome 2, position 17294203) was obtained from the Arabidopsis Biological Resource Center, and strains containing the insertion were identified using the PCR primers: *Arp* forward (“Arp4F”) 5′GATCGAAAGGAAATTGAGGCG3′ and Salk T-DNA *Lba1* 5′TGGTTCACGTAGTGGGCCATCG3′; wild-type genes were identified in separate reactions using Arp4F and Arp10.

### RNA analysis

RNA was extracted from young leaves or flowers by freezing ca 0.2 g of tissue in liquid nitrogen, grinding it with a sterilized pestle in a micro-centrifuge tube, and extracting it with Trizol (Gibco, Life Technologies, Grand Island, NY) following the manufacturer's procedure. Extracts were treated with 2 units of DNAase (“DNAfree”, Ambion, Applied Biosystems, 850 Lincoln Center Drive, Foster City, CA) at 37°C for 20 min, and the DNAase was removed according to the manufacturer's protocol. RT-PCR was performed using the Qiagen “One-Step” kit and the primers described above. In each case, the primer pair spanned an intron, so it would have been possible to detect DNA template contamination by the size of the amplified DNA. No DNA template was detected.

### Construction of multiple mutants

Transfer of pollen to emasculated stigmas was performed by standard methods. Germinated F1 seedlings were screened for the presence of the paternal allele by PCR using primers described above.

### Microscopy of embryos

Immature seeds were removed from siliques, fixed in a graded series of ethanol solutions, and cleared with methyl salicylate [9]. Cleared seeds were visualized with differential interference contrast optics.

## Results

**Figure 2:**
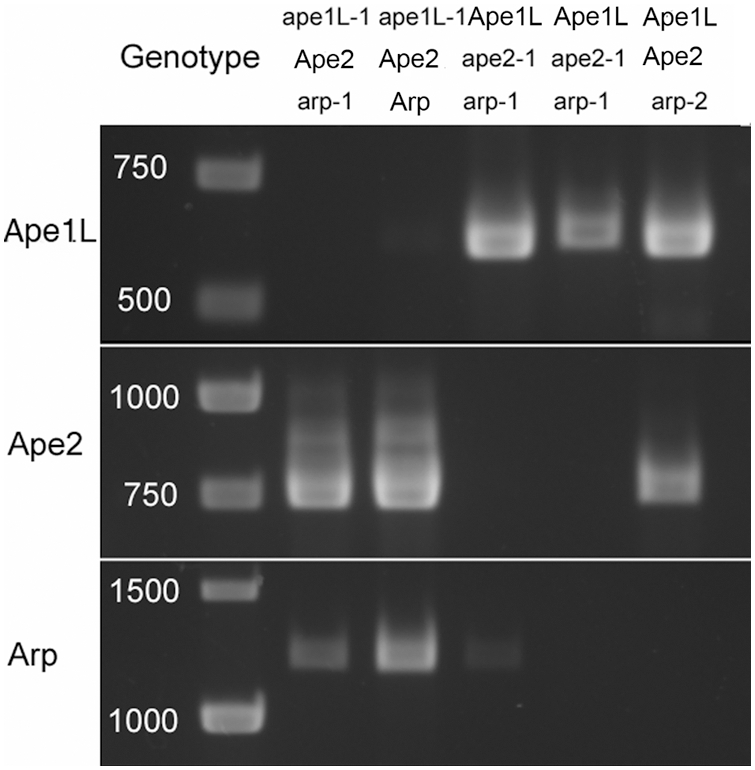
Expression of *Ape1L* (top), *Ape2* (middle), and *Arp* (bottom) genes. RT-PCR used template RNAs extracted from a selection of strains. Genotype of each strain, indicated by PCR analysis of extracted DNA, is shown at the top. In each strain with a T-DNA insertion, *ape1L-1*, *ape2-1*, and *arp-2*, accumulation of transcript of the corresponding gene was undetectable. RNA accumulation differed among the *arp-1* base-substitution strains.

The three genes, *Ape1L*, *Ape2*, and *Arp*, originally identified by homology to human abasic endonuclease genes, all code for proteins with amino acid homology to the xth/ exodeoxyribonuclease III family (Interpro: IPR004808; Fig. 1B). We identified mutants of all three genes (Fig. 1A) and combined the mutations through genetic crosses to determine which, if any, gene products were essential for viability under normal conditions. The homozygous *ape1L-1*, *ape2-1*, and *arp-2* strains, which contained T-DNA insertions, showed no detectable mRNA template, as assayed by RT-PCR (Fig. 2). Two of three *arp-1* lines, with the substitution mutation in *Arp*, showed lower, though still detectable, amounts of RT-PCR product, and one showed no detectable product (Fig. 2).

Strains containing a single mutation in any of the three genes, *ape1L-1*, *ape2-1*, *arp-1*, or *arp-2*, showed normal germination, growth, bolting and flowering, and no obvious effects on fertility. Triple heterozygotes were constructed by first crossing homozygous *ape2-1* to *arp-1* and self-crossing the progeny. The double homozygous mutant was identified by PCR; this mutant was then crossed to homozygous *ape1L-1*. F1 seeds from the resulting siliques were then planted, and the genotypes of the resulting plants were confirmed by PCR. The triply heterozygous plants were self-pollinated, and DNA extracts from the F2 progeny were analyzed by PCR. The results (Table 1) showed an absolute lack of progeny with homozygous double *ape1L-1/ape2-1* insertions. There was no negative relationship between the appearance of *ape1L-1* or *ape2-1* insertions and *arp-1* mutations.

**Table 1:** Genotypes of progeny from the self-cross of a plant triply heterozygous for *ape1L-1*, *ape2-1*, *and arp-1* and their wild-type alleles.^1^

Ape1L	Ape2	Arp	Observed	Expected
+/+	+/+	+/+	1	2.1
+/+	+/+	+/−	10	4.2
+/+	+/+	−/−	1	2.1
+/−	+/+	+/+	1	4.2
+/−	+/+	+/−	6	8.4
+/−	+/+	−/−	4	4.2
−/−	+/+	+/+	0	2.1
−/−	+/+	+/−	8	4.2
−/−	+/+	−/−	2	2.1
+/+	+/−	+/+	2	4.2
+/+	+/−	+/−	10	8.4
+/+	+/−	−/−	6	4.2
+/−	+/−	+/+	8	8.4
+/−	+/−	+/−	19	16.8
+/−	+/−	−/−	9	8.4
−/−	+/−	+/+	4	4.2
−/−	+/−	+/−	4	8.4
−/−	+/−	−/−	5	4.2
+/+	−/−	+/+	2	2.1
+/+	−/−	+/−	3	4.2
+/+	−/−	−/−	1	2.1
+/−	−/−	+/+	6	4.2
+/−	−/−	+/−	14	8.4
+/−	−/−	−/−	8	4.2
−/−	−/−	+/+	0	2.1
−/−	−/−	+/−	0	4.2
−/−	−/−	−/−	0	2.1
			Chi-square	40.2
			Confidence	P<0.05

1 Note the lack of progeny that are doubly homozygous for *ape1L-1* and *ape2-1* (last three rows).

To confirm the results, seeds from self-crosses of two strains, each heterozygous for *Ape1L/ape1L-1* and homozygous for *ape2-1* (and homozygous for either *Arp* or *arp-1*) were planted, and the seedlings were analyzed. In each case, no seedlings homozygous for both *ape1L-1* and *ape2-1* were found (Table 2A, B). These results indicated that in an *ape2* background at least one wild-type allele of *Ape1L* is essential for either gametogenesis or embryogenesis.

**Table 2:** Genotypes of progeny resulting from self-crosses of strains with various combinations of mutations in *Ape1L*, *Ape2*, and *Arp*.

*A. Ape1L^+/−^ape2^−/−^Arp^+/+^*		
*Ape1L* Progeny	Observed	Expected
+/+	15	10
+/−	25	20
−/−	0	10
	Chi-square	13.75
	Confidence	P<0.005

*B. Ape1L^+/−^ape2^−/−^arp^−/−^(arp-1)*		
*Ape1L* Progeny	Observed	Expected
+/+	14	11.25
+/−	31	22.5
−/−	0	11.25
	Chi-square	15.1
	Confidence	P<0.005

*C. Ape1L^+/−^ape2^−/−^arp^−/−^ (arp-2)*		
*Ape1L* Progeny	Observed	Expected
+/+	7	10
+/−	33	20
−/−	0	10
	Chi-square	19.35
	Confidence	P<0.005

*D. ape1L^−/−^Ape2^+/+^Arp^+/−^ (arp-2)*		
*Arp* Progeny	Observed	Expected
+/+	5	10
+/−	18	20
−/−	12	10
	Chi-square	3.70
	Confidence	NS

The preceding tests were conducted with strains containing the TILLING amino acid substitution mutants of *Arp*. Because it was possible that these mutants retained some ARP enzyme activity, we repeated the tests with the *arp-2* T-DNA insertion mutant. As in the previous tests (Table 1, Table 2A, B), no plants homozygous for both *ape1L-1* and *ape2-1* were found among the progeny of a self-cross of a plant of genotype *Ape1L/ape1L-1*, *ape2-1/ape2-1*, *arp-2/arp-2* (Table 2C). We observed no mutant phenotype associated with *arp-2* homozygosity. It was possible to find mutants homozygous for both *arp-2* and *ape2-1* (parents of the cross in Table 2C) and for both *arp-2* and *ape1L-1* (Table 2D). These results confirm that at least one of the *Ape1L* or *Ape2* genes must be present for seed viability, demonstrate that the *Arp* gene product does not compensate for a lack of both *Ape1L* and *Ape2* genes, and indicate that the *Arp* gene product is not itself required for seed viability.

**Figure 3:**
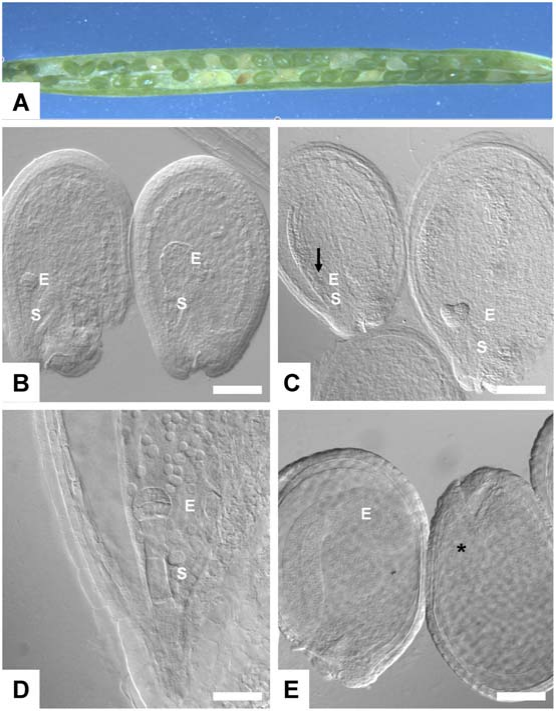
Development of *ape1L-1*, *ape-2-1* mutant seeds. A. Immature silique produced by self-cross of a plant heterozygous for *ape1L-1* and homozygous for *ape2-1*. B. Cleared pre-globular stage seeds showing one normally developing seed (left) and one aberrant (right) embryo proper and suspensor phenotype from the same silique; scale bar = 80 µm. A number of seeds continue to develop to the heart stage (C) while some seeds remain at the pre-globular stage of development (arrow); scale bar = 85 µm. D. Close-up of (C) showing a seed arrested at the pre-globular stage of development; scale bar = 15 µm. By the bent cotyledon stage (E) some seeds within the same silique show no visible embryo within the central vacuole (*); scale bar = 120 µm. E, embryo proper; S, suspensor.

Microscopic observation of mature green siliques from the *Ape1L/ape1L-1, ape2-1/ape2-1* self-crosses indicated that a significant fraction of seeds showed incipient or complete abortion. Healthy seeds were green; putatively aborted seeds were yellow, white, reddish, or collapsed (Fig. 3A). Quantitative analysis of the seeds in 23 siliques from *Ape1L/ape1L-1*, *ape2-1/ape2-1* plants gave 253 aborted seeds to 732 healthy seeds (ratio of 1∶2.9), consistent with the interpretation that the aborted seeds were *ape1L-1* homozygotes. Testing the seeds for germination gave the same result: of seeds from three siliques and two plants sown on nutrient Phytagel, 35 did not germinate; 116 germinated and grew to at least the two-true-leaf stage (ratio of 1∶3.0). In both cases the results were inconsistent with the hypothesis that the double mutation blocked female gametogenesis or gametophyte maturation (expected ratio 1∶1; Chi-square = 233 and 43, respectively; P<0.0001). They were similarly inconsistent with the hypothesis that the double mutation blocked pollination. They were consistent with embryo lethality.

In order better to understand the function of abasic endonucleases during seed development, we dissected siliques of the plants heterozygous for *ape1L-1* and homozygous for *ape2-1*. In cleared seeds of pre-globular stage siliques, most seeds contained octant embryos, although on rare occasions some of the seeds contained large embryos with aberrant cell number and size in both the embryo proper (E) and suspensor (S) (Fig. 3B). By the heart stage, the majority of seeds contained heart-shaped embryos (Fig. 3C), while some seeds remained at the pre-globular (octant) stage of development (Fig. 3D). As shown in Fig. 3E, cleared seeds at the bent cotyledon stage sometimes failed to show a visible embryo.

The phenotype was examined further by comparing the proportion of seeds arresting in the mutant lines (Table 3). When compared to control, many seeds of the cross between the heterozygous *ape1L-1* and homozygous *ape2-1* plants appeared to arrest before the heart stage of development. In mutant siliques at the heart stage (majority, 54%, of embryos at heart stage), a relatively large number of embryos remained in the pre-globular (18%) and globular (18%) stages of development. By comparison, in corresponding wildtype siliques, 94% of the embryos were at heart stage. At the bent cotyledon stage, a number of seeds contained visible pre-globular (12%) and globular (7.5%) stage embryos or no embryo at all (9.3%). In wildtype siliques of the same stage, 100% of the seeds had developed bent-cotyledon embryos. Taken together, the data indicate that an abasic endonuclease gene is essential for the progression of normal seed development after fertilization. Because abasic endonuclease may be provided to gametophytes by the parent sporophyte, the data do not exclude the possibility that abasic endonuclease function is important in gametophytes.

**Table 3:** Development of *ape1L-1*, *ape2-1* embryos in siliques at different stages of development^1^.

Embryo stages							
Silique stage^2^	Pre-globular	Globular	Heart	Torpedo	Bent cotyledon	No visible embryo	Malformed
Globular	30 (19)^3^	49 (79)	9.5 (3.0)	0	0	4.7 (0)	5.9 (0)
Heart	18 (0)	18(5.7)	54 (94)	1.4 (0)	0	7.4 (0)	1.4 (0)
Torpedo	13 (0)	8.6 (0)	4.3 (3.1)	61 (99)	4.3 (0)	8.6 (0)	0
Bent cotyledon	12 (0)	7.5 (0)	0	7.7 (0)	61 (100)	9.3 (0)	2.5 (0)

1 Siliques were taken from self-fertilized *ape1L-l^+/−^*, *ape2-1^−/−^* plants.

2 Silique stage was scored based on the stage of development of the largest class of embryos in the seeds of the silique.

3 Numbers indicate the proportion (%) of embryos of each stage found in mutant siliques; numbers in parentheses indicate the proportion (%) of seeds found in wild-type siliques at the same stage of development.

## Discussion

The results from self-crosses of heterozygous mutants of *Ape1L*, *Ape2*, and *Arp* show that full development of an embryo requires at least one wild-type copy of either *Ape1L* or *Ape2*. The statistics indicate that the genes function in the embryo sporophyte and that either a maternal or paternal copy of one wild-type allele is sufficient. Although the *Arp* gene product has abasic endonuclease activity [6], a wild-type allele of *Arp* does not compensate for the lack of *Ape1L* and *Ape2*. This is apparently not because of a lack of expression of *Arp* in embryos: *Arp* mRNA is present at approximately the same levels as *Ape1L* and *Ape2* mRNAs (M. Belmonte and J. Harada, NCBI gene expression omnibus accession number GSE11262).

Abasic endonucleases catalyze the second step in the base excision repair pathway. There are several ways to explain the requirement for base excision repair and the associated endonuclease in seed development. The pathway may function in the activation of essential genes through the programmed removal and replacement of specific bases in certain gene promoters; or it may play a more general role in protecting the genome from inactivation by replacing damaged bases. These roles are not mutually exclusive.

*Demeter* provides a model for the programmed involvement of base excision repair in normal plant development. Inactivation of the *Demeter* gene, which codes for a 5-methylcytosine glycosylase [10], [11], results in the inviability of the female gametophyte that inherits the gene [12]. The *Demeter* gene product is required for transcriptional activation of the *Medea* gene. An amino acid essential for this function is present in the glycosylase domain, suggesting that the glycosylase activity (and not just DNA binding activity) is involved in the activation of *Medea* [13]. Resynthesizing the promoter DNA through endonuclease, DNA polymerase, and DNA ligase functions replaces the 5-methyl-cytosines with cytosines and activates the *Medea* gene. If the Demeter glycosylase works through a base-excision and repair process, and if the loss of the *Dme* gene blocks development of the female gametophyte, one might ask why the loss of endonuclease genes does not block development at that same stage, an earlier stage than is actually observed. The answer is that the Demeter protein, like some other endonuclease III (nth)-related glycosylases, is dual function; it possesses a DNA lyase activity that cleaves the sugar-phosphate chain and may obviate the need for endonuclease activity [11]. The observations we report are consistent with the interpretation that Ape1L and Ape2 support a process of DNA modification following fertilization, one that depends on a mono-functional glycosylase and thus requires a separate endonuclease.

There are other cases that suggest that modification of DNA is important for plant seed development. The promoters of some barley hordein seed protein genes become hypomethylated at the stage when these proteins are synthesized [14], [15]. Defects in methylation affect size and viability of *Arabidopsis* seeds [16]–[18]. The role of abasic endonuclease and base excision repair in development may be of even more general significance—Wang et al. [19] found that inhibition of translation of mRNA from *abasic endonuclease 1* in zebra fish blocked embryonic development, and the authors suggested that the product of that gene was “regulating specific early stages.”

**Figure 4:**
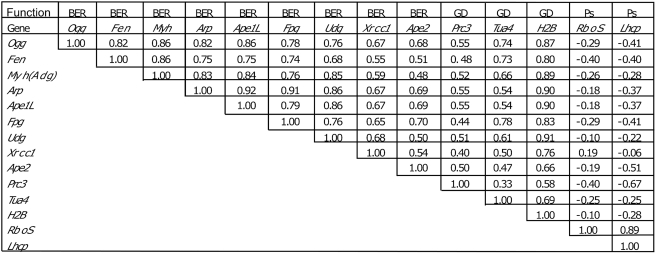
Correlations between genes related to DNA repair, growth and development, and photosynthesis. Correlations between the expression levels of genes in different organs and stages of development were calculated from the Arabidopsis Genome Expression data set in TAIR. Abbreviations: BER, genes of the base excision repair pathway: *OGG*, oxoguanine glycosylase (At1g21710); *Fen*, flap endonuclease (At5g26680); *Myh*, MutY homologue (adenine-DNA glycosylase, At4g12740); *Arp*, abasic endonuclease-redox protein (At2g41460); *Ape1L*, abasic endonuclease-1-like (At3g48425); *Fpg*, formamidopyrimidine-DNA glycosylase (At1g52500); *Udg*, uracil-DNA glycosylase (At3g18630); *XRCC-1*, X-ray repair complementing defective repair in Chinese hamster cells-1 (At1g80850); *Ape2*, abasic endonuclease 2 (At4g36050). GD: growth and development genes: *Prc3*, proteasome subunit a2 (At1g16470); *Tua4*, tubulin A4 (At1g04820); *H2B*, histone 2B (At1g07790). Ps: photosynthetic genes: *RboS*, ribulosebisphosphate small subunit (At1g67090); *Lhcp*, light harvesting chloroplast protein (At1g29910). The data set contains entries for 80 organs/stages, although not all entries have data for all genes. The 95% confidence level is ±0.18.

It is also likely that Ape1L and Ape2 participate in error-correcting base-excision repair. An analysis of levels of expression of relevant genes involved in base excision repair, using Expression Atlas of Arabidopsis Development (“AtGenExpress”) data from Affymetrix microarrays on the TAIR web site (www.arabidopsis.org) indicates strong correlations among base excision repair genes when their levels are compared in different *Arabidopsis* organs and stages of development (Fig. 4). For instance, the correlation coefficients between expression values for *Fpg* and *Arp*, *Ape1L*, and *Ape2* are 0.91, 0.79, and 0.70. The correlations are also high between expression values for the base excision repair genes and three genes associated with growth and development, genes for proteasome subunit a2, tubulin A4, and histone 2B. In contrast, the correlation coefficients between expression values for each of these genes and two genes related to photosynthesis in mature leaves, *RboS* (small subunit of rubisco) and *Lhcp* (light harvesting chloroplast protein), are negative. This supports the idea that the Arp, Ape1L, and Ape2 endonucleases function with other base excision repair enzymes to protect genome integrity in dividing cells, but not in the cells of mature tissues. The importance of the endonucleases at the sporophytic stage of seed development could be dependent on their repair function if base modifications are particularly prevalent at that stage. The finding that embryo development apparently slows or stops at various stages of development, rather than at one particular stage (Table 3), supports this hypothesis. Abasic endonucleases that are essential for development under conditions producing base modifications have been found in other organisms. The loss of *apn1* in yeast makes the cells hypersensitive to agents such as MMS and H_2_O_2_, which alkylate or oxidize DNA bases [20]–[22]. Antisense reduction of *ape1* expression in human cells increases their sensitivity to DNA damage [23].
